# Universal elastic-hardening-driven mechanical instability in *α*-quartz and quartz homeotypes under pressure

**DOI:** 10.1038/srep10810

**Published:** 2015-06-23

**Authors:** Juncai Dong, Hailiang Zhu, Dongliang Chen

**Affiliations:** 1 Beijing Synchrotron Radiation Facility, Institute of High Energy Physics, Chinese Academy of Sciences, Beijing 100049, China

## Abstract

As a fundamental property of pressure-induced amorphization (PIA) in ice and ice-like materials (notably *α*-quartz), the occurrence of mechanical instability can be related to violation of Born criteria for elasticity. The most outstanding elastic feature of *α*-quartz before PIA has been experimentally reported to be the linear softening of shear modulus *C*_44_, which was proposed to trigger the transition through Born criteria *B*_3_. However, by using density-functional theory, we surprisingly found that both *C*_44_ and *C*_66_ in *α*-quartz exhibit strong nonlinearity under compression and the Born criteria *B*_3_ vanishes dominated by stiffening of *C*_14_, instead of by decreasing of *C*_44_. Further studies of archetypal quartz homeotypes (GeO_2_ and AlPO_4_) repeatedly reproduced the same elastic-hardening-driven mechanical instability, suggesting a universal feature of this family of crystals and challenging the long-standing idea that negative pressure derivatives of individual elastic moduli can be interpreted as the precursor effect to an intrinsic structural instability preceding PIA. The implications of this elastic anomaly in relation to the dispersive softening of the lowest acoustic branch and the possible transformation mechanism were also discussed.

Pressure-induced amorphization (PIA) along with related phenomena of density- and entropy-driven polyamorphism has provided enormous insights into the metastable behavior of materials under pressure^1^,^2^. However, owing to the non-equilibrium nature of those transitions, their actual occurrence and evolution can depend upon many factors (e.g., kinetics, non-hydrostaticity, and interpretation techniques), and hence understanding the underlying physics remains challenging. In particular, one of the long-standing debates is about the overarching conceptual framework for PIA in network-forming oxides, notably ice and silica^3^,^4^,^5^. Those systems are typically formed by polyhedral building blocks at low pressures and possess significant capacity for densification; besides, they have been usually associated with several unusual features such as negative melting lines and/or negative thermal expansion. On the basis of extensive experimental studies and theoretical simulations, four microscopic mechanisms for interpreting PIA have been proposed^2^, including thermodynamical melting^6^,^7^, mechanical melting^3^,^8^,^9^, steric constraints^10^, and ferroelastic glasses^11^. However, up to date no clear-cut experimental demonstration in favor of a particular mechanism has been established. In ice I*h*, recent neutron scattering study reported that PIA could be caused by a crossover of thermodynamical melting and mechanical melting where phonon softening plays a central role^12^.

To resolve the above dilemma, a practical approach is to closely examine the structural transformation pathways and the modification of the complex configurational energy landscape (CEL) under pressure, which can be captured by the changes of elastic constants and phonon dispersion relations^13^. In *α*-quartz, various structural evolution models have been proposed to be responsible for the destabilization process^14^,^15^,^16^,^17^,^18^,^19^,^20^. Meanwhile, high-pressure elasticity has been extensively examined theoretically, with serious discrepancy^21^,^22^,^23^; experimental studies are, however, extremely scarce since complete measurement of the elastic moduli remains challenging. While *α*-quartz was reported to amorphize at pressures of around 18–35 GPa^24^, Brillouin spectroscopy study on single-crystal *α*-quartz to above 20 GPa revealed that Born stability criteria *B*_3_ is violated at an extrapolated pressure of 39 GPa, which was shown to be driven by softening of *C*_44_^5^. Vanishing of individual elastic constant, which usually complies with phonon softening at Brillouin zone center, has been confirmed in other compounds undergoing PIA such as ice I*h*^9^ and B_4_C^25^, and is thereafter widely interpreted as the precursor effect to lattice instability for PIA. Based on this result, attempts to explain the microstructure of the amorphous SiO_2_ exhibiting elastic anisotropy and memory effects have been made^11^. However, when ultrasonic method was used, most of the measurements have obtained a positive pressure derivative of *C*_44_ at low pressures (<1 GPa) for *α*-quartz^26^,^27^. A *B*_2_-type instability was claimed, whereas another instability was given by the decreasing of *C*_66_ with pressure. Lately, by using density-functional theory (DFT), a reinvestigation of all the elastic constants of *α*-quartz indicated that the measured elasticity by Brillouin scattering at higher pressures is incompatible with the available x-ray diffraction (XRD) data of the volumetric and axial compressibility^28^. Unlike ice^9^, lattice instability in quartz and quartz-like materials was predicted to be initiated by acoustic phonon softening at Brillouin zone edge^29^,^30^, which was thereby supposed to precede elastic instability, but an improved DFT calculation exhibited that the zone-edge frequency and a combined “elastic constants” vanish almost simultaneously^23^. The questions regarding how elastic instability is induced and how far phonons are involved in PIA hence remain controversial. Here we are encouraged to further investigate these issues by a unified DFT simulation of the elastic and dynamical behaviors of quartz homeotype family (SiO_2_, GeO_2_, and AlPO_4_) under pressure, and some parallels and contrasts between our results and those previously obtained in ice are also discussed.

## Results and Discussion

Local density approximation (LDA) usually underestimates the equilibrium structural parameters at ambient pressure (see Supplementary Table S1); however, the calculated pressure evolution of the volumetric and anisotropic axial compressibility, with *a* axis being more compliant than *c* axis, agree with experimental values extremely well, as contrast to the large underestimation by generalized gradient approximation (see Supplementary Fig. S1). Moreover, the predicted deformation of tetrahedral units and frequencies of long-wavelength phonons are in excellent agreement with XRD and Raman data, respectively (see Supplementary Figs. S2, S3, S4), confirming that LDA has an ability to model the compression of quartz and quartz homeotypes accurately.

**Figure 1:**
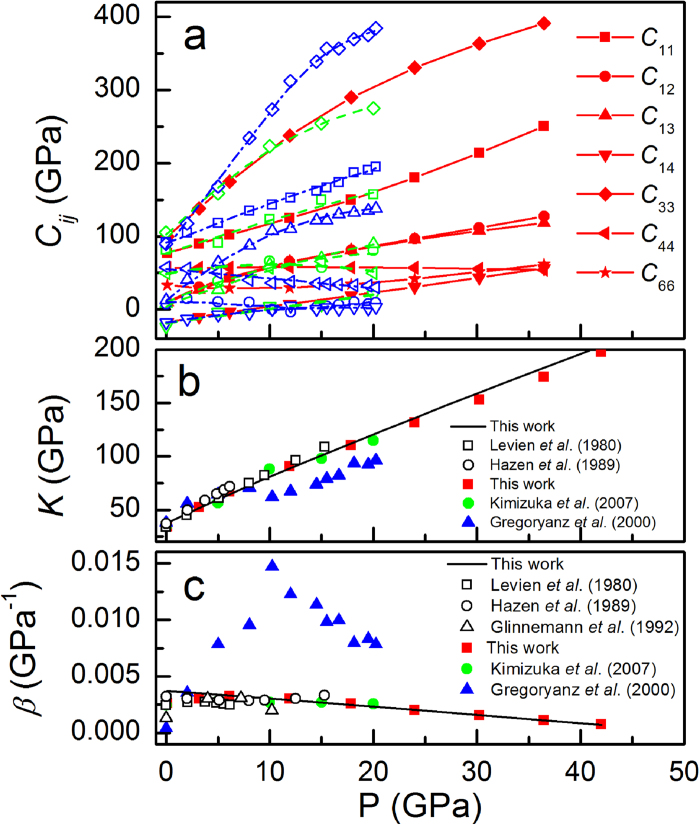
Elastic properties of α-quartz at high pressures. (**a**) Individual elastic constants, (**b**) bulk modulus, and (**c**) the anisotropy of linear compressibility of *α*-quartz SiO_2_ as a function of pressure. In (**a**) the solid-curve penetrated symbols represent this work, the dash-curve penetrated symbols LDA calculation^28^, and the dot-dash-curve penetrated symbols Brillouin scattering experiment^5^. In (**b**) and (**c**) the solid squares, solid circles^28^, and solid triangles^5^ are calculated from elastic moduli. The solid lines, open squares^43^, open circles^15^, and open triangles^44^ are calculated from the volumetric and axial compressibility data.

The obtained elastic moduli *C*_ij_ of *α*-SiO_2_ as a function of pressure (see also Supplementary Table S2) are shown in Fig. 1a. At ambient pressure, our calculated *C*_ij_ are consistent with the experimental values, whereas large discrepancies emerge at elevated pressure. We find that all the moduli tend to increase monotonously with pressure except *C*_44_ and *C*_66_ which show strong nonlinearity. *C*_44_ first increases with pressure up to about 12 GPa and then decreases gradually under further compression, as opposed to the decreasing of *C*_66_ up to about 6 GPa and then bending up. Ultrasonic measurements reported a positive pressure derivative for *C*_44_ and a negative pressure derivative for *C*_66_ at pressures of lower than 1 GPa^26^,^27^, while, by using Brillouin scattering, Gregoryanz *el al*. obtained a negative pressure shift of *C*_44_ and a positive pressure shift of *C*_66_ at all of the pressures to above 20 GPa^5^. Furthermore, there exists large difference in the magnitudes, especially for *C*_44_, *C*_12_, and *C*_33_ (see Fig. 1a). By linear extrapolation, the soft *C*_44_ derived from Brillouin scattering would vanish at 39 GPa, but our calculation indicates that *C*_44_ will not approach zero until around 140 GPa. The experimental value of *C*_12_ shows weak pressure dependence with magnitude close to zero; however, similar increases of *C*_12_ and *C*_13_ are observed in our calculation. An amount of 80 GPa is shown for the discrepancy of *C*_33_ beyond 20 GPa. Similar trends have been observed in recent DFT studies^23^,^28^. It is noteworthy that *C*_14_ changes sign near 9 GPa and intersects with *C*_44_ near 36 GPa. The pressure evolutions of *C*_ij_ for *α*-GeO_2_ and *α*-AlPO_4_ (see Supplementary Table S2, Figs. S5 and S6) resemble the case in *α*-SiO_2_ that evidences their mechanical analogy. To our knowledge, this is the first report of the high-pressure dependence of the complete set of elastic constants for those two compounds.

The validity of the obtained *C*_ij_ as a function of pressure in a trigonal crystal can be generally checked through the bulk modulus (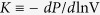) and the anisotropy of linear compressibility (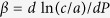)^28^, since they can be not only related to the volumetric and axial compressibility data, but also expressed in terms of the combination of *C*_ij_,
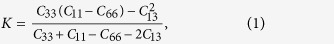

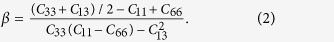


Figure 1b,c shows, respectively, the comparison of the derived values of *K* and *β* based on the two methods. The results yielded from *C*_ij_ of present study (solid square) and recent DFT calculation (solid circle) are sufficiently compatible with the calculated volumetric and axial compressibility values (solid lines) as well as XRD data (open symbols), whereas those obtained from Brillouin scattering (solid triangle) are severely off the lines at high pressures. It confirms the consistency between our *C*_ij_ values and the compressibility data, but also implies that the experimental *C*_ij_ at high pressures may require numerical reexamination as pointed out by Kimizuka *et al*^28^. Reasonable agreement is observed in the cases of *α*-GeO_2_ and *α*-AlPO_4_ (see Supplementary Figs. S5 and S6). The discrepancy in various XRD data might be due to the origin and nature of samples and the degree of non-hydrostaticity.

The mechanical stability of a homogeneously strained crystal can be captured through the Born stability criteria^31^, which are associated with the positive-definiteness of the elastic constant tensor. For a trigonal crystal, these criteria give rise to three necessary conditions, which expressed in the Voigt notation are
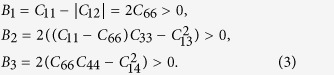


**Figure 2:**
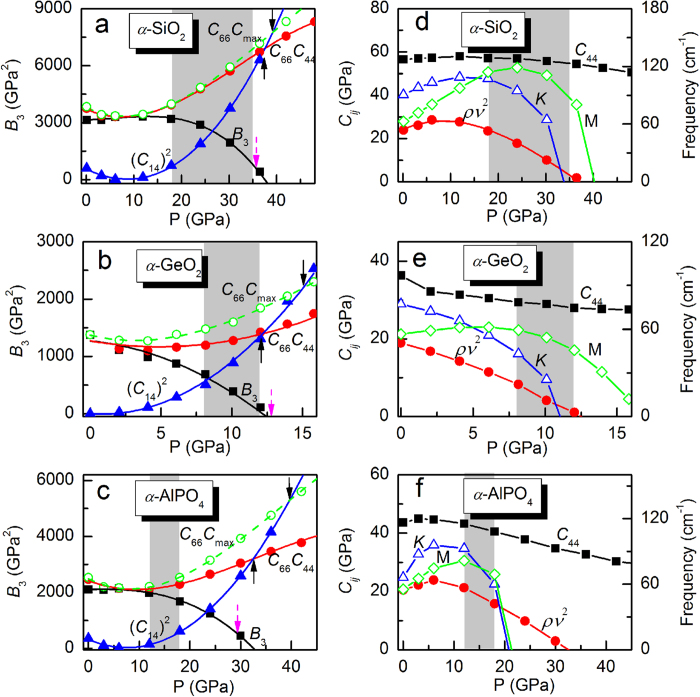
Born coefficient *B*_3_ and soft acoustic mode elastic constants. (**a**)–(**c**) Born coefficient *B*_3_ and (**d**)–(**f**) soft acoustic mode elastic constant *ρν*^2^ of *α*-SiO_2_, *α*-GeO_2_, and *α*-AlPO_4_ as a function of pressure. In (**a**)–(**c**), the respective contributions of *C*_66_*C*_44_ (solid circles) and 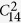 (solid triangles) to *B*_3_ (solid squares) are shown. The open circles are 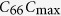 with 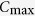 being the maximum value of *C*_44_. The dashed arrows represent the pressures where *C*_44_ crosses with *C*_14_. In (**d**)–(**f**), the variation of *C*_44_ and the calculated zone-edge *K*- and *M*-point soft mode frequencies with pressure are also displayed. The gray areas indicate the amorphization boundaries determined from the experiments^20^,^24^,^36^.

Figure 2a,b,c and Supplementary Fig. S7 display the computed pressure dependence of these coefficients. For *α*-SiO_2_, *B*_1_ and *B*_2_ both increase with pressure, while *B*_3_ decreases rapidly beyond 18 GPa and become negative around 38 GPa. Similar behavior for the three Born criteria is observed in the reported experimental results^5^ and theoretical studies^22^,^23^. Brillouin scattering experiment suggested *B*_3_ = 0 at 39 GPa, in comparison with about 30 GPa given by first-principles calculations. Nevertheless, there are vital differences in the determination of what triggers the mechanical instability. First-principles calculation by Binggeli *et al*^22^ predicted that neither *C*_66_ nor *C*_44_ would vanish in the pressure region of amorphization and thus vanishing of *C*_66_ or *C*_44_ is not the cause of instability. On the contrary, Brillouin scattering study by Gregoryanz *el al*.^5^ revealed that the violation of *B*_3_ is triggered by softening of *C*_44_, because they found that *B*_3_ and *C*_44_ would approach zero around the same pressure. For convenience, we show in Fig. 2a the respective contributions of *C*_66_*C*_44_ and 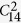 to *B*_3_ obtained from present DFT study. They both go through a minimum near 10 GPa and then increase, but 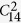 has a much greater pressure derivative than *C*_66_*C*_44_ above 18 GPa. The effect of *C*_44_ can be unveiled by constraining it to a constant (see Fig. 2a): the resulting pressure of *B*_3_ = 0 only shows a small positive shift of about 1 GPa, meaning that stiffening of *C*_14_ prevails over softening of *C*_44_. Surprisingly, it indicates that the violation of *B*_3_ is dominated by hardening of *C*_14_; the cases of *α*-GeO_2_ and *α*-AlPO_4_ are similar (see Fig. 2b,c), thereby strongly suggesting that the mechanical instability driven by stiffening of *C*_14_ may be a universal feature of quartz-like materials. Of special interest is the crossover pressures of *C*_14_ and *C*_44_ (dashed arrows in Fig. 2a,b,c), which are close to that of *B*_3_ = 0 and thus can serve as a good estimate for elastic instability. The very fact of that the reported beginning pressures of PIA in quartz and quartz-like compounds by various experiments (gray areas in Fig. 2a,b,c) generally correspond to the onset of the rapid decrease of *B*_3_ confirms that the elastic stability actually defines a homogeneous upper limit for the crystalline phase to persist.

**Figure 3:**
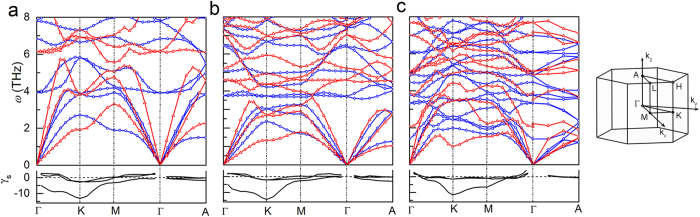
Phonon dispersions and mode Grüneisen parameters at high pressures. Top: Calculated phonon dispersions of (**a**) *α*-SiO_2_ at 0 GPa (circles) and 30 GPa (triangles), (**b**) *α*-GeO_2_ at 0 GPa (circles) and 8 GPa (triangles), and (**c**) *α*-AlPO_4_ at 0 GPa (circles) and 20 GPa (triangles). Bottom: Mode Grüneisen parameters of the transverse and longitudinal acoustic branches at 30 GPa for *α*-SiO_2_, 8 GPa for *α*-GeO_2_, 20 GPa for *α*-AlPO_4_. The Brillouin zone of the trigonal lattice is shown at the right.

The elastic instability implies softening of acoustic phonon occurring at infinitesimal ***q*** wave vector along high-symmetry directions. Indeed, in trigonal crystal, the slopes of the transverse acoustic branches close to the Brillouin zone center in the Γ-*A* and Γ-*M* directions correspond respectively to the elastic constants of *C*_44_ and *C*_66_, whereas those in the Γ-*K* direction are proportional to a combination of elastic constants, namely 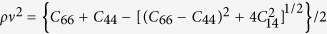
27. Lattice dynamics was investigated, with the full phonon dispersions at various pressures shown in Fig. 3. Our ambient pressure data for *α*-SiO_2_ and *α*-AlPO_4_ reproduce well the low-energy part of their phonon dispersions as given by inelastic neutron scattering studies^32^,^33^. As pressure increases, contrary to the hardening of almost all the optical modes, pronounced softening happens at the lowest acoustic modes in the Γ-*K* direction. The transverse acoustic branches close to the zone center in the Γ-*M* direction show obvious hardening, whilst those in the Γ-*A* direction decrease slightly. These results signal a negative pressure dependence for *ρν*^2^ and *C*_44_ and a positive for *C*_66_ in this pressure range, which coincides well with the tendency as unveiled by the stress-strain relations. Under further compression, the entire portion of the acoustic branch goes soft (see Supplementary Fig. S8). To examine the precursor effect of the elastic instability to the acoustic branch softening, Fig. 2d,e,f exhibits the variations of *ρν*^2^, *C*_44_, and the frequencies of the zone-edge *K* and *M* points as a function of pressure. Because *B*_3_ is given by the product of *ρν*^2^ with 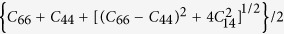, *ρν*^2^ becomes negative at the pressures of *B*_3_ = 0, which are soon after the first instability at the *K* point but before the instability at the *M* point except in *α*-AlPO_4_ where the frequencies of the *K* and *M* points becomes imaginary almost simultaneously. It contradicts the result of recent DFT calculation by Choudhury *et al*^23^ that *B*_3_ = 0 happens nearly at the same pressure of the *K*-point instability, which may be owing to the less accuracy of their calculation. *C*_44_ only shows slight decreasing in the pressure regime. Therefore, we argue that the symmetry-adapted combination of *C*_ij_ (*ρν*^2^ or *B*_3_) rather than the individual elastic constant (*C*_66_ or *C*_44_) allows for suitable prediction of the lattice instability in quartz homeotypes. The approximate linearity and negative slope of *ρv*^2^ in the higher pressure regime was interpreted as an indicator of proper ferroelastic behavior for the phase transition^5^. Because the vanishing of a single vibrational mode is indicative of a mechanical instability towards a low-symmetry ordered phase, the collapse of the entire portion of an acoustic branch at higher pressures in quartz and quartz homeotypes suggests the competition between many phases, which may manifest macroscopically as a disordered low-symmetry structure.

**Figure 4:**
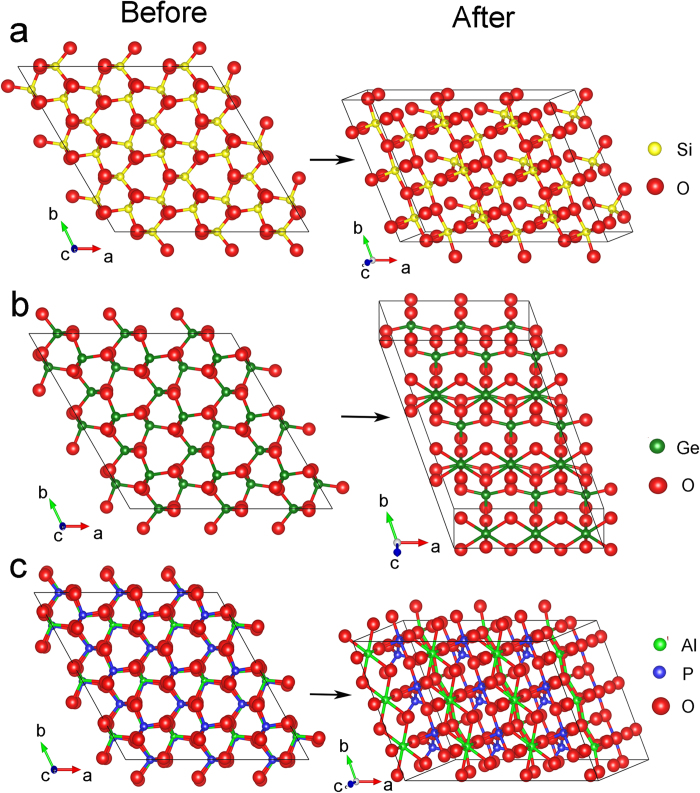
Structural changes commensurate with the soft *K*-point wave vector. Structural changes of (**a**) SiO_2_, (**b**) GeO_2_, and (**c**) AlPO_4_ from the initial *α* phase to the modulated phases at respectively 40, 12, and 36 GPa commensurate with the reciprocal lattice vector (1/3, 1/3, 0).

To understand the transformation mechanism, the deformation behaviors of the three compounds before and after the instability were analyzed. Just before the vanishing of *K*-point mode, the six closing intertetrahedral angles and the two cross opening intratetrahedral angles intersect with each other (see Supplementary Fig. S3), indicating that cooperative rotations of tetrahedral units result in flattening of themselves due to nearest-neighbor intertetrahedral anion-anion repulsions^20^, which just creates low-energy passageways for cations to move from tetrahedral to octahedral bonding. Inspection of the topology of the opening intratetrahedral angles reveals a spiral order parallel to the *c* direction (see Supplementary Fig. S3), which may play an important role in originating the stiffening of most of the elastic moduli and the elastic instability. It appears like that the spiral order is strongly correlated with the emergence of a high-symmetry anion packing, as evidenced by the converging *x*, *y*, and *z* fractional coordinates (see Supplementary Fig. S2). The nature of the interatomic arrangement resulting from the instability was investigated by structural optimizations on a 3 × 3 × 1 supercell (commensurate with the *K*-point wave vector) where atoms were displaced from equilibrium along a pattern corresponding to the soft *K*-point mode as the starting configuration. It is shown that when the cations are shifted to the edges of the tetrahedral units under further compression, a shear instability in *xy* plane occurs, leading to the lateral movement of the anions with respect to each other until a denser packing containing octahedrally coordinated cations forms (see Fig. 4). Particularly, *α*-SiO_2_ transforms to quartz II phase (space group *C*2) with alternating layers of tetrahedral and octahedral configurations, whereas *α*-GeO_2_ to a monoclinic phase (space group *C*2/*m*) containing only GeO_6_ octahedra. The resulting structure for AlPO_4_ consists in AlO_6_ octahedra, PO_4_ tetrahedra, and PO_6_ octahedra, which can be artifact as PO_6_ octahedra was not observed until above near 80 GPa^34^. Those obtained post-quartz phases qualitatively reproduce the experimental observations. The transformation pathways share the same eutaxic ordering conjectured by O’Keeffe *et al*^35^, which emphasizes the balance between attractive and repulsive forces among ions, while simultaneously maximizing the density. However, consistent with the dynamical instability revealed for both cations and anions by partial phonon density of states (see Supplementary Fig. S9), the cations and anions are found to respond to pressure cooperatively rather than moving in a sequential fashion as proposed by Huang *et al*^19^. The resulting structures show different coordination environment, indicating that the transformation pathway should depend crucially on the strength of the covalent bonding, which might explain the mixed coordination configurations observed in SiO_2_ and AlPO_4_, instead of in GeO_2_. On releasing pressure, only the low-symmetry phase in AlPO_4_ reverts to the quartz-like phase, similar to the experimental observation of “memory effect” in *α*-AlPO_4_ rather than in *α*-SiO_2_ and *α*-GeO_2_^34^.

Valuable insights into PIA can be obtained by comparison with ice. Strong analogies exist between amorphization in *α*-quartz and in ice. In hexagonal ice I*h*, violation of Born criteria was reported, accompanied by flattening of the entire lowest transverse acoustic branch^9^. However, with *C*_14_ equal to zero, one difference with respect to *α*-quartz is that the violated Born criteria *B*_2_ of ice I*h* is proportional to *C*_66_ which exhibits dramatic decreasing under compression and thus can be considered as the driving force for the elastic instability. Also, *C*_66_ is associated with the phonon softening in the Γ-*M* direction, supporting the precursor effect of individual soft elastic constant to lattice instability before amorphization transition. Another difference with respect to *α*-quartz is that the lowest transverse acoustic branch in ice I*h* shows an almost dispersionless flattening that starts at the Brillouin zone center. With phonon softening initiated at the *K*-point zone edge, the mode Grüneisen parameters 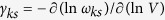 of quartz and quartz homeotypes at high pressures, however, show an obvious dispersion relation (see the bottom part of Fig. 3). It has been pointed out that where the soft phonon modes start might be irrelevant^3^. However, as the large number of soft phonons and their relations actually reflect the nature of the complex CEL topography of the accessible phase space, we argue that PIA would depend on the fashion by which phonon branch collapses. It can be apparently modulated by factors such as chemical effect, strain fluctuations, thermal activation, and impurity, thus resulting in various transformation pathways. Under homogeneous compression, the local energy minima corresponding to the soft *K*-point phonon is preferred, which can be the explanation for some recent studies which indicate that in the case of *α*-quartz and several quartz homeotypes, such as GeO_2_ and *AB*O_4_ (*A* = Al, Ga, Fe; *B* = P, As) berlinites, the previously observed high-pressure amorphous structure may be crystalline^24^,^36^. In the presence of strongly, heterogeneous stress, the amorphous materials may consist in a mixture of two or more competing disordered phases and thus exhibit elastic anisotropy^24^ due to domains of the poorly crystallized structures. In AlPO_4_, the memory effect^34^ might be explained as the reversibility of the crystalline-to-crystalline phase transitions. The PIA might also take place, with the high-density amorphous form described as a dense array of oxygen ions where the cations are disordered over the octahedral sites^37^.

## Conclusions

In summary, although many previous experimental and theoretical studies have reported the violation of Born stability criteria in *α*-quartz and quartz homeotypes (GeO_2_ and AlPO_4_), the present work constitutes the first unified *ab initio* investigation on this issue, with a general picture obtained. It has revealed a strong nonlinearity for both *C*_44_ and *C*_66_ and an elastic instability triggered by stiffening of *C*_14_, instead of by decreasing of *C*_44_. Compelling evidences have been provided. This universal elastic-hardening-driven mechanical instability has settled the long-standing controversy about the microscopic mechanism of *α*-quartz between ultrasonic and Brillouin scattering experiments (where Born criteria at higher pressures were usually derived from simple linear extrapolation of *C*_ij_), while in turn challenging the original idea that negative pressure derivatives of individual elastic moduli can be interpreted as the precursor effect to an intrinsic instability in those trigonal structures preceding PIA. On the other hand, our calculations have shown a dispersive softening of the entire lowest acoustic branch, which contrasts drastically with the dispersionless softening of Ice I*h* and implies that the actual occurrence of amorphization in quartz-like materials might rely on the degree of heterogeneous stress. We also note that the amorphization mechanism in Ice I*h* was reported to be temperature-dependent^12^, and whether this phenomenon occurs in *α*-quartz remains to be classified, which can lead to a deeper understanding of PIA in network-forming oxides. These findings would have broad implications for understanding questions ranging from the metastability and densification mechanisms of network-forming oxides under pressure to the geological processes in the Earth’s mantle, glass formation, or developing high-toughness ceramics.

## Methods

The structure of *α*-SiO_2_ (*α*-GeO_2_) consists of a network of corner-linked SiO_4_ (GeO_4_) tetrahedra which are arranged in virtual threefold left-handed helices running parallel to the *c* axis, the overall symmetry being trigonal (*P*3_2_21). *α*-AlPO_4_ adopts a similar arrangement with AlO_4_ and PO_4_ tetrahedra alternatively interconnected. Our DFT calculations were performed by using Quantum ESPRESSO package^38^ with the LDA exchange-correlation functional^39^. All electron-ion interaction was described by norm-conserving, optimized, designed nonlocal pseudopotentials, generated with the OPIUM code^40^. The electronic wavefunctions were expanded in a plane-wave basis set with a kinetic energy cutoff of 100 Ry and the Brillouin zone was sampled in Monkhorst-Pack *k* point meshes with an interpolation grid spacing of 0.03 Å^−1^, to achieve the total energy convergence of less than 0.1 mRy/atom. Structural optimizations were performed by using BFGS quasi-newton algorithm. The effective elastic constants (i.e., Birch coefficients) *C*_*ij*_ as a function of pressure^5^,^28^ were obtained via the stress-strain relations^41^, while phonon frequencies were calculated by linear response method based on perturbation theory^42^.

## Additional Information

**How to cite this article**: Dong, J. *et al* Universal elastic-hardening-driven mechanical instability in a-quartz and quartz homeotypes under pressure. *Sci. Rep* **5**, 10810; doi: 10.1038/srep10810 (2015).

## Supplementary Material

Supplementary Information
