# Supplementary material for: Universal elastic-hardening-driven mechanical instability in α-quartz and quartz homeotypes under pressure
Source: Sci Rep. 2015 Jun 23;5:10810. doi: 10.1038/srep10810 (PMC4477368; doi:10.1038/srep10810)
Supplement: Supplementary Information [file srep10810-s1.pdf]

Supplementary Information for

Universal elastic-hardening-driven mechanical instability in α-quartz and quartz homeotypes under pressure

Juncai Dong, Hailiang Zhu, and Dongliang Chen*

Beijing Synchrotron Radiation Facility, Institute of High Energy Physics, Chinese Academy of Sciences, Beijing 100049, China

**E-mail: chendl@ihep.ac.cn*

Supplementary Figure S1

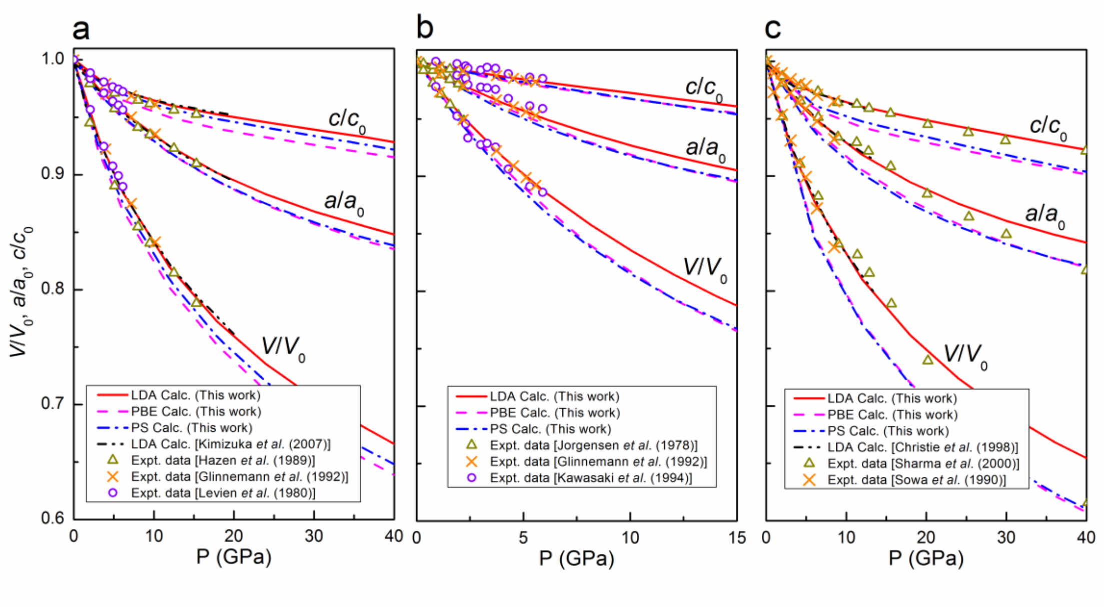

Supplementary Figure S1. Pressure evolution of the calculated (full line) lattice parameters and unit-cell volumes of (a) α-SiO_2, (b) α-GeO_2, and (c) α-$AlPO_4$, in comparison to reported local density approximation (LDA) calculations[1, 2] and experimental data[3, 4, 5, 6, 7, 8, 9] (symbols). The generalized gradient approximation with the Perdew-Burke-Ernzerhof (PBE)[10] functional and its version optimized for solids (PBEsol)[11] were also calculated for comparison.

Supplementary Figure S2

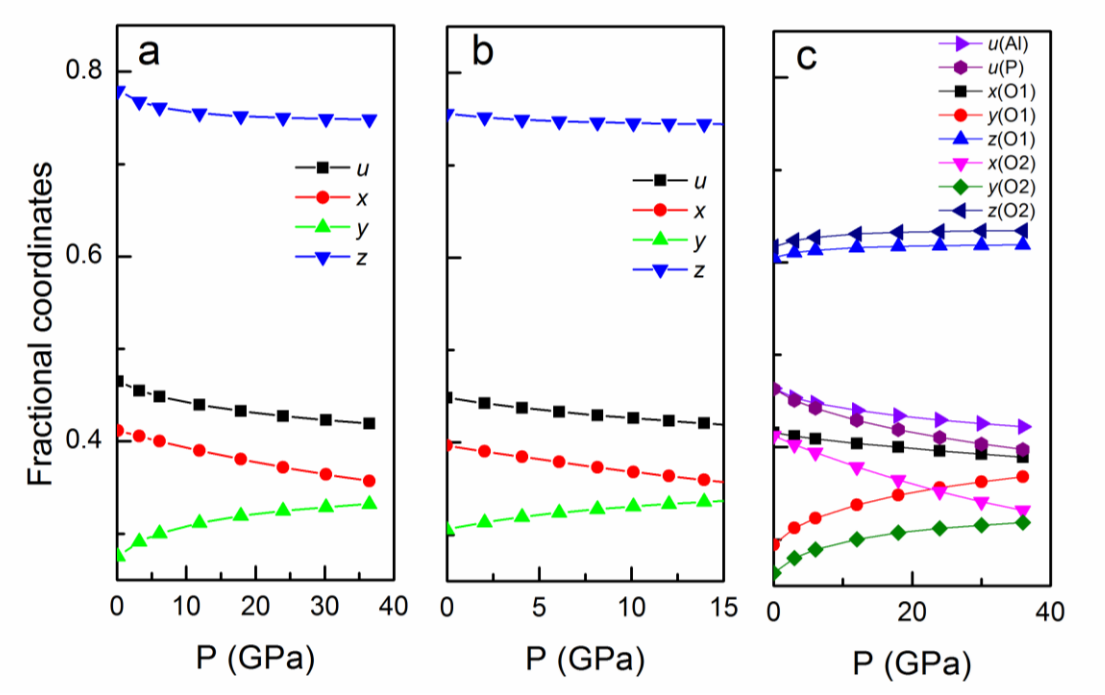

Supplementary Figure S2. Fractional coordinates (*u*, *x*, *y*, and *z*) of the *α* phase unit cell of (a) SiO_2, (b) GeO_2, and (c) $AlPO_4$ as a function of pressure.

Supplementary Figure S3

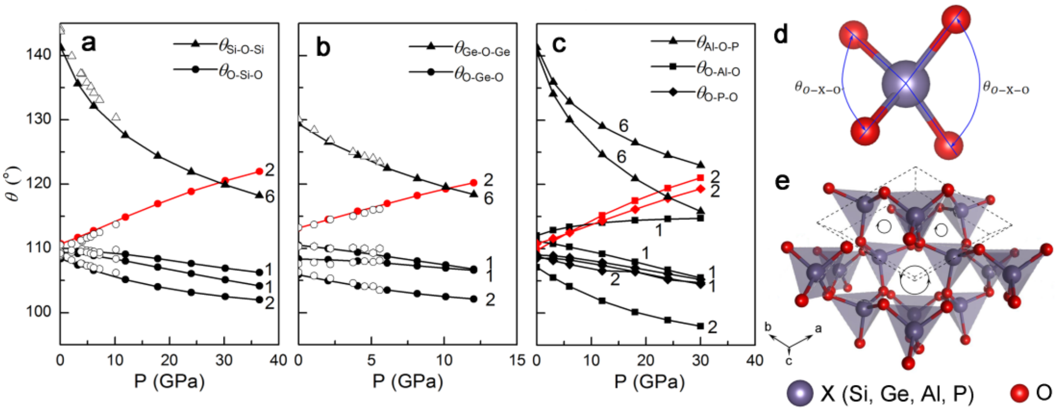

Supplementary Figure S3. Evolution of the intertetrahedral X-O-X angles (X = Si, Ge, Al, P) and the intratetrahedral O-X-O angles of the *α* phase unit cell of (a) SiO_2, (b) GeO_2, and (c) $AlPO_4$ with pressure. The open symbols are from Refs. 4, 5, 12. The numbers represent the degeneracy of the bond angles. The two opening intratetrahedral angles are cross to each other as shown in (d). The topology of the opening intratetrahedral angles, which reveals a spiral order parallel to the *c* direction, is shown in (e). They are of opposite handedness with respect to the sixfold double helices (double arrows) of XO_4 (X = Si, Ge, Al, P) tetrahedra.

Supplementary Figure S4

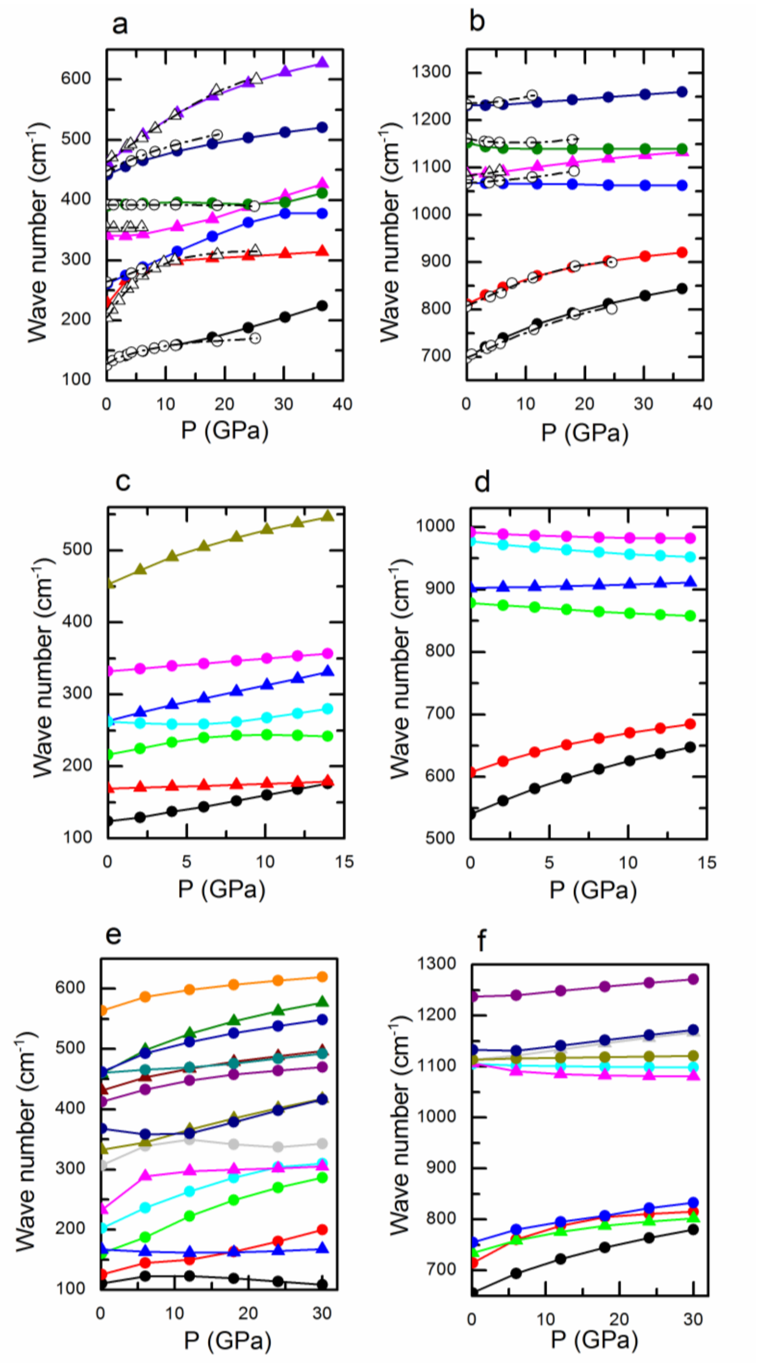

Supplementary Figure S4. Long wavelength phonon frequencies of (a)-(b) α-SiO_2, (c)-(d) α-GeO_2, and (e)-(f) α-$AlPO_4$ as a function of pressure. The circles represent the E modes and the triangles the A_1 modes. The solid symbols correspond to calculated values of this work, and the open symbols to Raman data from Ref. 13.

Supplementary Figure S5

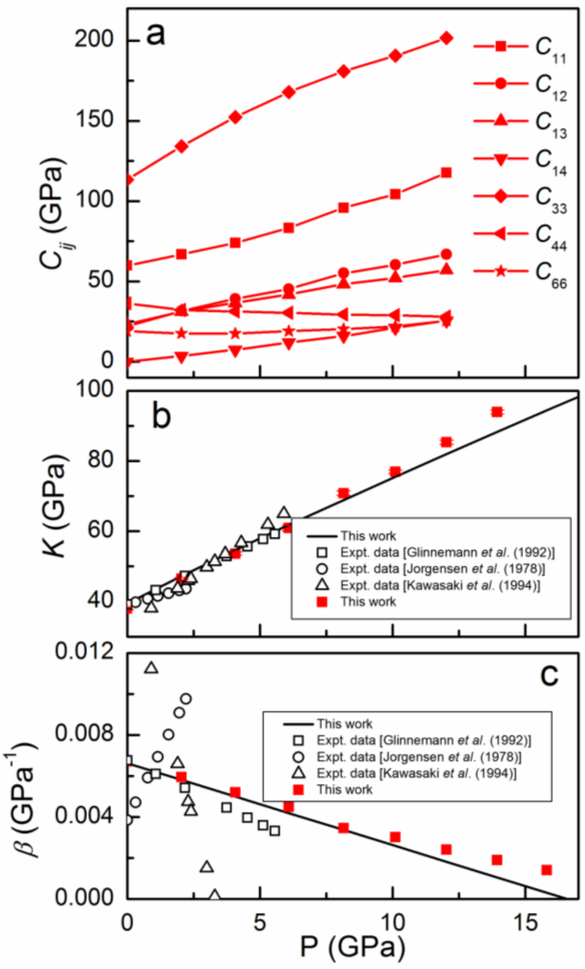

Supplementary Figure S5. (a) Individual elastic constants, (b) bulk modulus, and (c) the anisotropy of linear compressibility of α-GeO_2 as a function of pressure. In (b) and (c), the solid squares are calculated from elastic moduli. The solid lines, open squares[4], open circles[6], and open triangles[7] are calculated from the volumetric and axial compressibility data.

Supplementary Figure S6

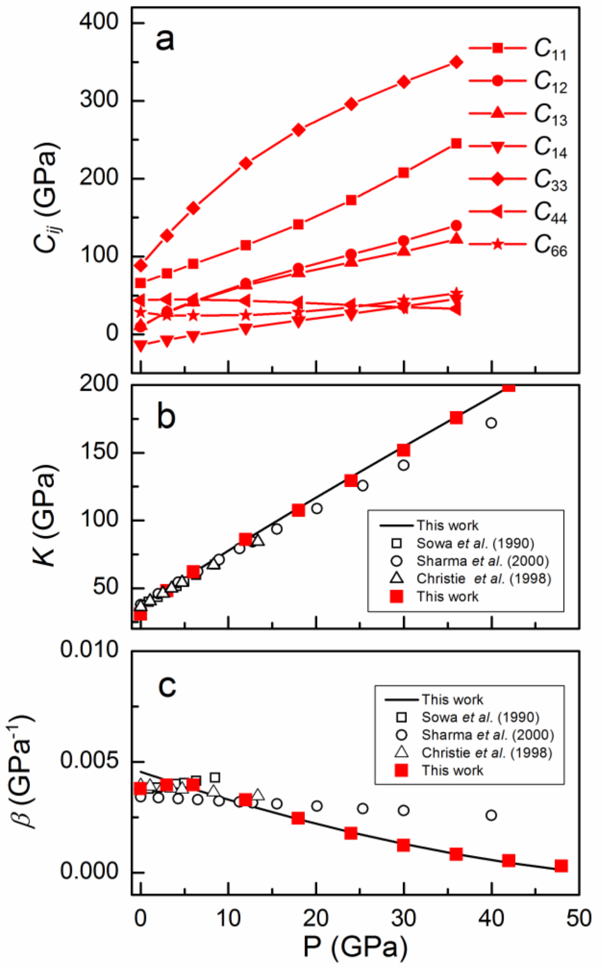

Supplementary Figure S6. (a) Individual elastic constants, (b) bulk modulus, and (c) the anisotropy of linear compressibility of α-$AlPO_4$ as a function of pressure. In (b) and (c), the solid squares are calculated from elastic moduli. The solid lines, open squares[9], open circles[8], and open triangles[2] are calculated from the volumetric and axial compressibility data.

Supplementary Figure S7

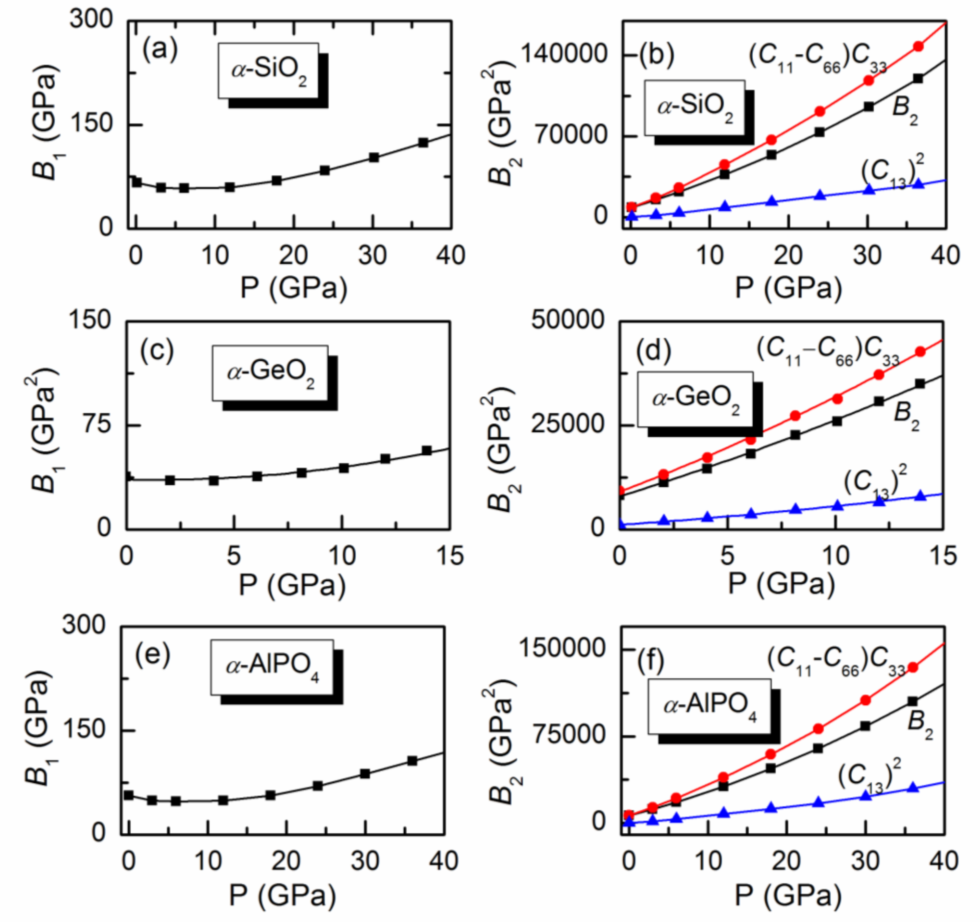

Supplementary Figure S7. Pressure dependence of the Born coefficients B_1 and B_2 of α-SiO_2, α-GeO_2, and α-$AlPO_4$. The respective contributions of $(C_{11}-C_{66})C_{33}$ (circles) and C_{13}^2 (triangles) to B_2 (squares) are shown.

Supplementary Figure S8

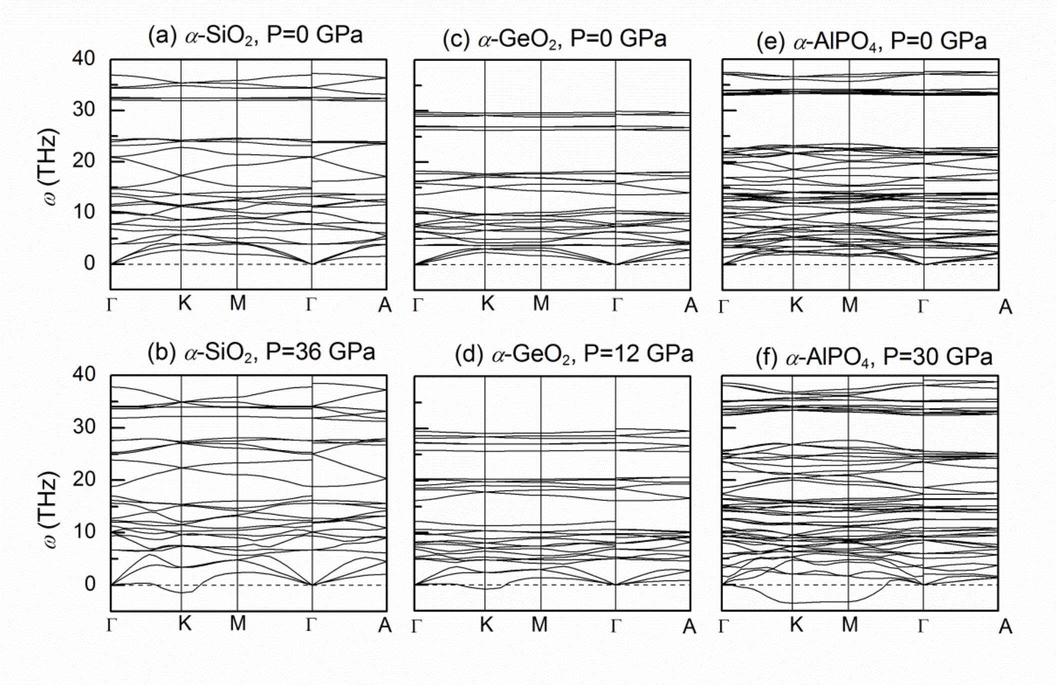

Supplementary Figure S8. The computed high pressure phonon dispersion relations of α-SiO_2, α-GeO_2, and α-$AlPO_4$.

Supplementary Figure S9

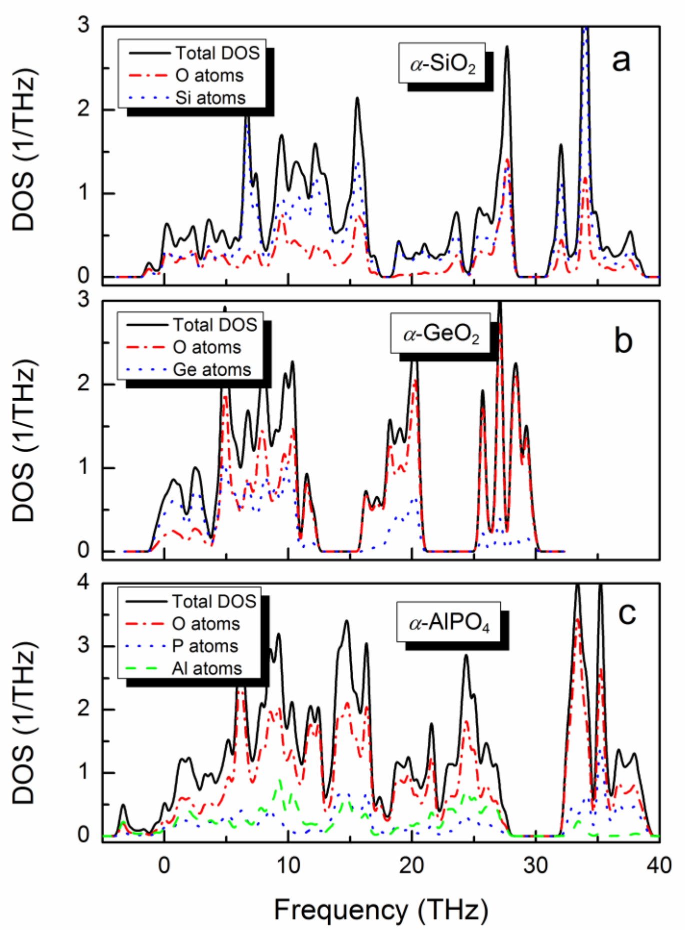

Supplementary Figure S9. Total and partial phonon density of states (DOS) of (a) α-SiO_2 at 36 GPa, (b) α-GeO_2 at 12 GPa, and (c) α-$AlPO_4$ at 30 GPa. The partial phonon density of states shows that the imaginary frequencies originate from both the lattice dynamics of the central cations (Si, Ge, Al) and the corner O atoms in the tetrahedral scheme, implying that the instability should be taking place by means of a cooperative movement between cations and oxygen sublattice.

Supplementary Table S1

Supplementary Table S1. Calculated equilibrium lattice parameters (a_0 and b_0 in Å), bulk modulus (K_0 in GPa) and its pressure derivatives (K_0') by three-order Birch-Murnaghan equation of state for α-SiO_2, α-GeO_2, and α-$AlPO_4$, together with experimental and theoretical values for comparison.

Compound	Functional	Lattice constant		Bulk modulus	
		a_0	b_0	K_0	K_0'
α-SiO_2	LDA	4.8525	5.3494	37.3	4.75
		4.863[a]	5.364[a]	37.7[a]	4.9[a]
	PBE	5.0282	5.5168	36.3	4.16
	PBEsol	4.9514	5.4511	37.0	4.25
	Expt.	4.914[b]	5.406[b]	34[b]	5.7[b]
		4.9148[c]	5.4062[c]	36.4[c]	6.3[c]
		4.921[d]	5.4163[d]		
α-GeO_2	LDA	4.8811	5.5517	39.6	3.88
	PBE	5.1015	5.7707	33.4	2.72
	PBEsol	5.0126	5.7283	36.1	3.22
	Expt.	4.9851[c]	5.6455[c]	39.1[c]	2.2[c]
		4.9844[d]	5.6477[d]	39.2[d]	3.8[d]
α-$AlPO_4$	LDA	4.8497	10.7409	35.0	4.6
		4.9414[e]	10.947[e]	36.3[e]	4[e]
	PBE	5.0835	11.2059	26.8	4.4
	PBEsol	5.0192	11.0971	25.2	4.7
	Expt.	4.9386[f]	10.959[f]	37.88[f]	4[f]
		4.941[g]	10.94[g]	36[g]	4[g]

[a]LDA calculation from Ref. 1.

[b]Experimental data from Ref. 3.

[c]Experimental data from Ref. 6.

[d]Experimental data from Ref. 4.

[e]LDA calculation from Ref. 2.

[f]Experimental data from Ref. 8.

[g]Experimental data from Ref. 9.

Supplementary Table S2

Supplementary Table S2. Effective elastic moduli (C_{ij} in GPa), bulk modulus (K in GPa), and the anisotropy of linear compressibility (β in 10^{-3} GPa^{-1}) of α-SiO_2, α-GeO_2, and α-$AlPO_4$ at high pressures (C_{12}=C_{11}-2C_{66}). Previous experimental and theoretical results at 0 GPa are included for comparison.

		P=0 GPa			6 GPa	12 GPa	18 GPa	24 GPa	36 GPa
α-SiO_2		Expt.[a]	Expt.[b]	Calc.	Calc.	Calc.	Calc.	Calc.	Calc.
	C_{11}	87.2	86.8	76.7	101.8	124.9	149.9	180.3	250.7
	C_{12}	7.2	7.0	10.3	43.7	65.6	81.0	96.5	127.1
	C_{13}	11.9	11.9	9.7	41.8	64.7	80.7	95.2	118.3
	C_{14}	-17.8	-18.0	-17.4	-3.3	7.7	19.2	30.7	56.1
	C_{33}	105.5	105.8	99.6	174.8	237.9	289.8	330.5	391.1
	C_{44}	58.7	58.2	56.6	57.3	58.0	57.2	57.0	54.4
	C_{66}	40.0	39.9	33.2	29.0	29.7	34.4	41.9	61.8
	K	37.7	37.4	34.3	66.9	90.7	110.5	131.7	174.4
	β	2.38	2.43	2.62	3.24	3.04	2.59	2.03	1.10

		P=0 GPa			2 GPa	4 GPa	6 GPa	8 GPa	12 GPa
α-GeO_2		Expt.[c]	Calc.[d]	Calc.	Calc.	Calc.	Calc.	Calc.	Calc.
	C_{11}	66.4	64	59.8	66.8	74.1	83.3	95.8	117.7
	C_{12}	21.3	22	21.9	31.6	39.1	45.3	55.2	66.9
	C_{13}	32.0	32	23.3	31.2	36.6	41.9	48.3	57.0
	C_{14}	-2.2	-2	-0.2	3.5	7.5	12.0	15.9	25.6
	C_{33}	118.0	118	113.3	134.2	152.4	168.0	180.9	201.6
	C_{44}	36.8	37	36.3	32.2	31.4	30.5	29.4	27.9
	C_{66}	22.5	21	18.9	17.6	17.5	19.0	20.3	25.4
	K	42.4	42	38.0	46.5	53.6	60.9	70.9	85.4
	β	7.5	8	6.71	5.96	5.21	4.50	3.45	2.41

		P=0 GPa			6 GPa	12 GPa	18 GPa	24 GPa	36 GPa
α-$AlPO_4$		Expt.[e]	Calc.[f]	Calc.	Calc.	Calc.	Calc.	Calc.	Calc.
	C_{11}	64.9	87.9	65.8	90.3	114.3	141.1	172.5	245.3
	C_{12}	8.9	27.1	9.4	42.3	65.2	84.7	102.5	139.6
	C_{13}	14.6	30.4	10.8	41.6	63.1	78.6	92.6	122.0
	C_{14}	-12.1	-11.2	-13.4	-1.4	8.4	17.5	26.5	45.6
	C_{33}	87.1	122.4	88.7	162.3	219.9	262.8	296.0	349.9
	C_{44}	43.1	43.3	43.7	44.7	43.3	40.6	37.8	32.7
	C_{66}	27.9	30.4	28.2	24.0	24.5	28.2	35.0	52.8
	K	31.7	51.3	30.7	62.1	85.9	107.5	129.4	175.8

β	4.6	3.1	3.8	4.0	3.3	2.5	1.8	0.83

[a]Experimental data from Ref. 14.

[b]Experimental data from Ref. 15.

[c]Experimental data from Ref. 16.

[d]Experimental data from Ref. 17.

[e]Experimental data from Ref. 18.

[f]Theoretical values from Ref. 19.

References

1. Kimizuka, H., Ogata, S., Li, J. & Shibutani, Y. Complete set of elastic constants of α-quartz at high pressure: a first-principles study. *Phys. Rev. B* **75**, 054109-054114 (2007).

2. Christie, D. M. & Chelikowsky, J. R. Structural properties of α-berlinite ($AlPO_4$). *Phys. Chem. Miner.* **25**, 222-226 (1998).

3. Hazen, R. M., Finger, L. W., Hemley, R. J. & Mao, H. K. High-pressure crystal chemistry and amorphization of α-quartz. *Solid State Commun.* **72**, 507-511 (1989).

4. Glinnemann, J., *et al.* Crystal structures of the low-temperature quartz-type phases of SiO_2 and GeO_2 at elevated pressure. *Z. Kristallogr.* **198**, 177 (1992).

5. Levien, L., Prewitt, C. T. & Weidner, D. J. Structure and elastic properties of quartz at pressure. *Am. Mineral.* **65**, 920-930 (1980).

6. Jorgensen, J. D. Compression mechanisms in α-quartz structures-SiO_2 and GeO_2. *J. Appl. Phys.* **49**, 5473 (1978).

7. Kawasaki, S., Ohtaka, O. & Yamanaka, T. Structural change of GeO_2 under pressure. *Phys. Chem. Miner.* **20**, 531-535 (1994).

8. Sharma, S. M., Garg, N. & Sikka, S. K. High-pressure x-ray-diffraction study of α-$AlPO_4$. *Phys. Rev. B* **62**, 8824-8827 (2000).

9. Sowa, H., Macavej, J. & Schulz, H. The crystal structure of berlinite $AlPO_4$ at high pressure. *Z. Kristallogr.* **192**, 119 (1990).

10. Perdew, J. P., Burke, K. & Ernzerhof, M. Generalized gradient approximation made simple. *Phys. Rev. Lett.* **77**, 3865-3868 (1996).

11. Perdew, J. P., *et al.* Restoring the density-gradient expansion for exchange in solids and surfaces. *Phys. Rev. Lett.* **100**, 136406 (2008).

12. Yamanaka, T. & Ogata, K. Structure refinement of GeO_2 polymorphs at high pressures and temperatures by energy-dispersive spectra of powder diffraction. *J. Appl. Crystallogr.* **24**, 111-118 (1991).

13. Hemley, R. J. Pressure Dependence of Raman Spectra of SiO_2 Polymorphs: α-Quartz, Coesite, and Stishovite in *High-Pressure Research in Mineral Physics: A Volume in Honor of Syun-iti Akimo*to (ed. Manghnani, M. H. & Syono, Y.) 347-359 (American Geophysical Union, 2013).

14. Calderon, E., *et al.* Complete determination of the elastic moduli of *α*-quartz under hydrostatic pressure up to 1 GPa: an ultrasonic study. *J. Phys.: Condens. Matter* **19**, 436228 (2007).

15. McSkimin, H. J., Andreatch, P. & Thurston, R. N. Elastic moduli of quartz versus hydrostatic pressure at 25 ° and -195.8 ℃. *J. Appl. Phys.* **36**, 1624-1632 (1965).

16. Grimsditch, M., Polian, A., Brazhkin, V. & Balitskii, D. Elastic constants of *α*-GeO_2. *J. Appl. Phys.* **83**, 3018 (1998).

17. Balitskii, D. B., *et al.* Elastic, piezoelectric, and dielectric properties of *α*-GeO_2 single crystals. *Crystallogr. Rep.* **45**, 145-147 (2000).

18. Kramer, G. J., Farragher, N. P., van Beest, B. W. H. & van Santen, R. A. Interatomic force fields for silicas, aluminophosphates, and zeolites: derivation based on ab initio calculations. *Phys. Rev. B* **43**, 5068-5080 (1991).

19. Labéguerie, P., Harb, M., Baraille, I. & Rérat, M. Structural, electronic, elastic, and piezoelectric properties of α-quartz and MXO_4 (M=Al, Ga, Fe; X=P, As) isomorph compounds: a DFT study. *Phys. Rev. B* **81**, 045107 (2010).
